# When relief is out of reach: living with chronic itch

**DOI:** 10.1093/skinhd/vzaf053

**Published:** 2025-06-23

**Authors:** Geoffrey Hanley, Keavy Conroy, Sarah Fleming, Eilis Ni Chinneide, Clare Harnett

**Affiliations:** Department of Dermatology, Beaumont Hospital, Dublin, Ireland; Department of Dermatology, Beaumont Hospital, Dublin, Ireland; Department of Dermatology, Beaumont Hospital, Dublin, Ireland; Department of Dermatology, Beaumont Hospital, Dublin, Ireland; Department of Dermatology, Beaumont Hospital, Dublin, Ireland

## Abstract

A case of a gentleman living with chronic itch in the setting of systemic mastocytosis with cutaneous involvement.

## Patient viewpoint

Systemic mastocytosis was not a term I was in any way familiar with before my diagnosis. For years I suffered with one symptom – itch. This was not a regular itch, and I found it difficult to describe just how this felt and how it could dominate my life to such an extent.

**Figure 1:**
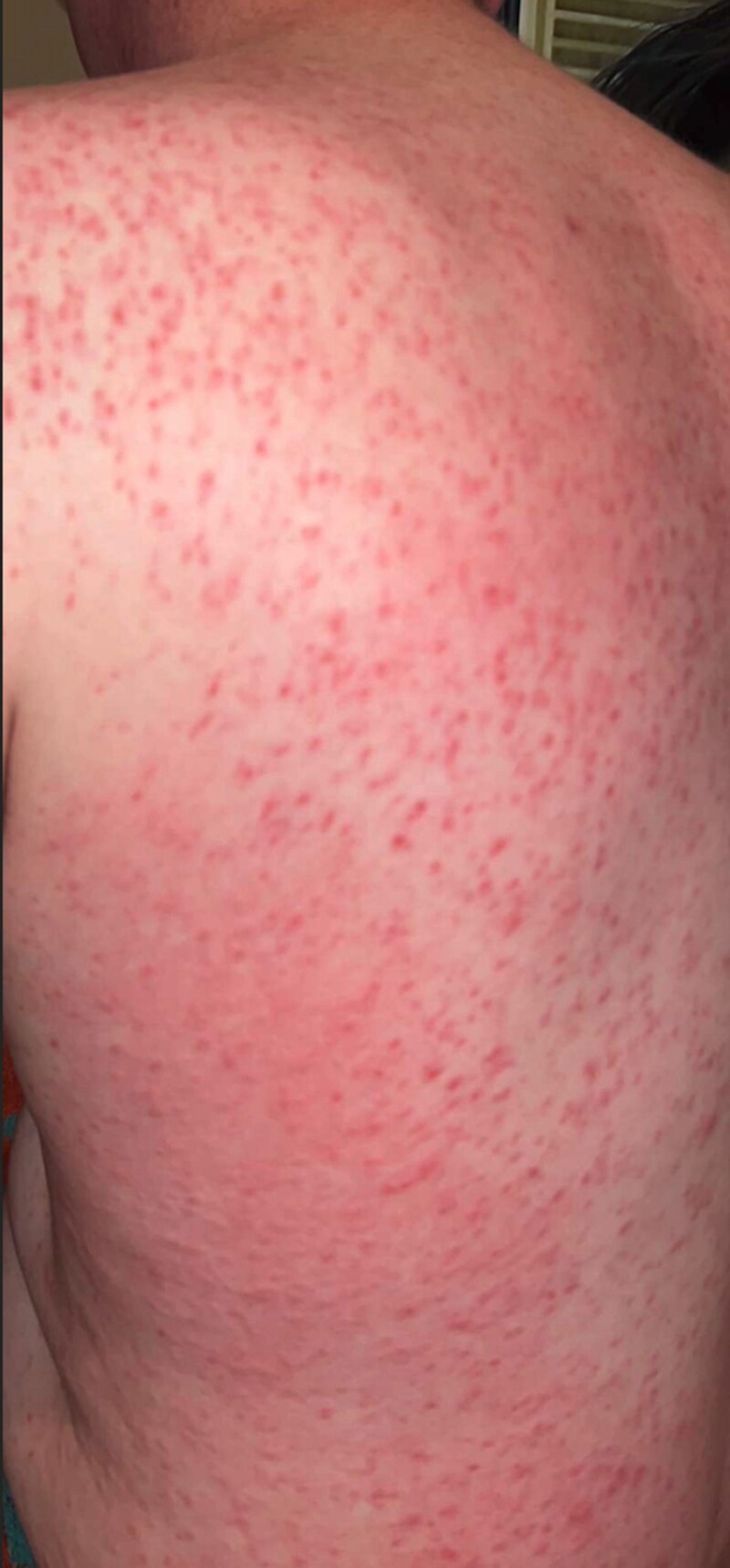
Maculopapular, urticarial rash on the back during flare of mastocytosis.

**Figure 2:**
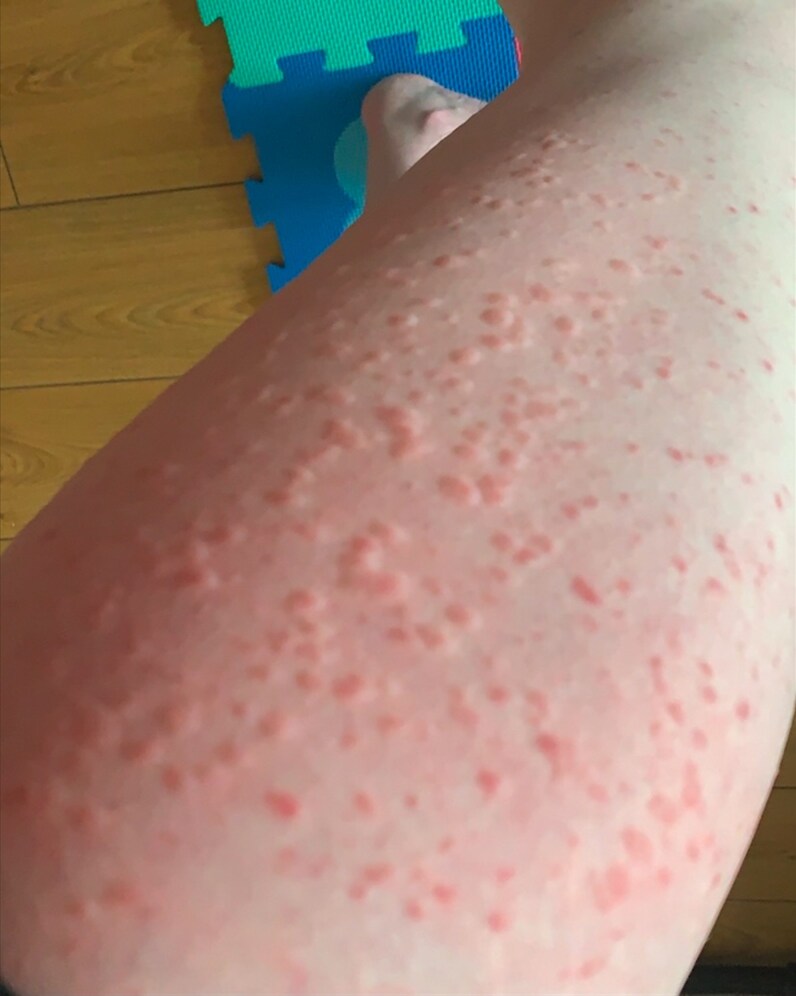
Maculopapular, urticarial rash on the leg during flare of mastocytosis.

**Figure 3:**
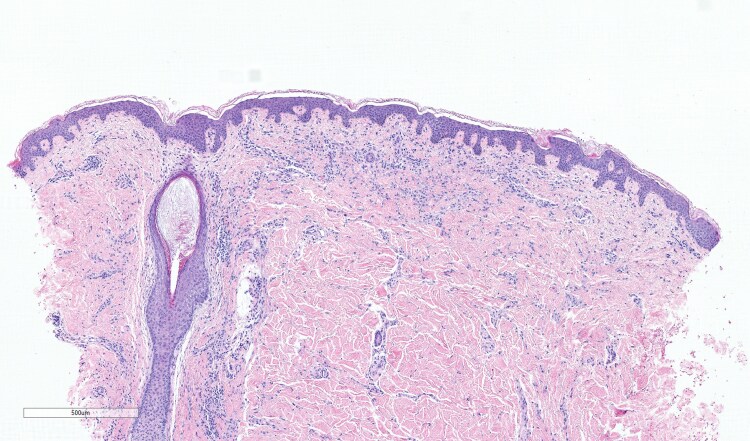
Skin biopsy (haematoxylin and eosin, total magnification ×4; scale bar = 500 µm) showing mast cell infiltrates in dermis (purple stained).

**Figure 4:**
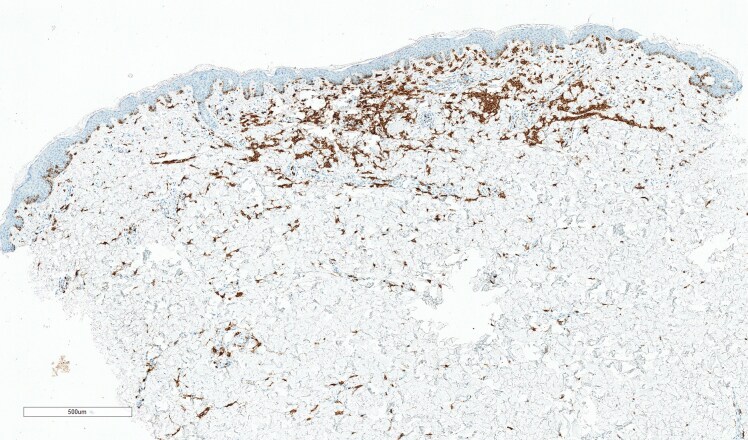
Skin biopsy (CD117 stain, total magnification ×4; scale bar = 500 µm) showing CD117 positively stained mast cells (brown stained), a marker of mast cell disease.

I lived most of my life without a label on my condition in a rural part of Ireland. For many years I was told I had a diagnosis of eczema simply because I was red and itchy (Figures 1, 2). In my twenties, something changed in my body and the itch was indescribable. To add to this misery, symptoms of diarrhoea and abdominal cramps became apparent and I knew it was time to see my doctor. My diagnosis was finally suggested when I attended a fragile bones clinic after being reviewed in the Emergency Department. For context, I had suffered multiple low impact fractures from benign activities like lifting a friend which fractured my spine, or bumping gently into my car steering wheel which broke multiple ribs. It was not until the combination of these fractures (which were later revealed as osteoporotic fractures), my rashes and my gastrointestinal symptoms were noted by a doctor and the suspicion of mastocytosis was raised. Eventually, after seeing multiple specialities, it was arranged for me to have a panel of investigations. Bloods, genetic tests, and biopsies of my skin (Figures 3, 4) and bones were arranged quickly, and elevated tryptase levels, alongside two biopsies with genetic testing, confirmed this wretched diagnosis. Accepting the diagnosis I can appreciate would be a very difficult process for a lot of people, but I had suffered a lot of trauma in my life and I felt like I was mentally steadfast to handle news like this.

When people think of being itchy I think they are at least relieved by the excitement of the scratch and relief of doing so. For me, the itch I felt was always an inch too far, like I was encased in a rubber wetsuit and nothing was sharp enough or deep enough to tackle the itch. But it is more than just regular itch, it is a burning sensation, the best comparison I can think of is the feeling of being stung by nettles, a raw, sensitive feeling with a tingling associated with it.

Sometimes the symptoms were unpredictable, the itching would feel like a burning, prickling sensation, other times like a furnace where I would have to remove my clothes, even in public settings, for relief of the heat. I describe myself being relieved of the symptoms as ‘allowing my skin to breathe’.

Triggers of the itch were just as unforgiving for me. I could be cold or hot, moving or immobile, being clothed or being showered, and my symptoms would rage. Wool material is unbearable, cotton is best, but all could trigger on a bad day.

I can appreciate itch is not a deadly symptom, but being told I was able to live a full life and work and so forth, was of little comfort. My mood, sleep and irritability caused me to withdraw from others. I would mostly avoid social events, my best remedy is distraction of any sort. My Playstation, TV subscriptions and most of all my one-year-old baby have thankfully been excellent distractions.

For me, distracting from itch was a full-time job. Applying creams and taking tablets are helpful, but never really felt like they were addressing the source of the problem. As if the anxiety was not enough, being constantly drowned in greasy emollients or coconut oil was never really a convenient or sustainable practice, especially as I worked as a mechanic where slippery hands are not exactly sought after in the profession.

Behavioural strategies have been somewhat helpful but mostly frustrating. I have tried things like breathing techniques and meditation but unfortunately substances, such as cannabis, were self-medicating techniques I gravitated towards. As time went on tended to use more and more cannabis to help with my itch. I felt I had nowhere else to turn. I avoid alcohol due to the effect is has on my mood.

Chronic itch is more than a physical ailment, it is exhausting, and affects my ability to focus on other important things. Every time an area is scratched it seems to be replaced by another more aggressive patch, an impossible to win scenario. This has made me depressed, anxious and lonely at times, but I am not someone who would ever consider acting on these feelings.

Much to my disappointment, there was no cure for this condition but I have learned to take it day by day, and appreciate little victories. I am grateful that my doctors have been able to treat me with Rydapt (midostaurin) but I feel discontented knowing that a more advanced medication known as Ayvakyt (avapratinib) is still not available to me at time of writing.

Living with this disease is an ongoing journey, of resilience, adaptation and trying to find the silver lining on the darker days. Nevertheless, I am thankful to my hospital and team of doctors who work tirelessly for me and will hopefully one day give me the gift of itch-free skin.

## Clinician’s comments

**Figure 5:**
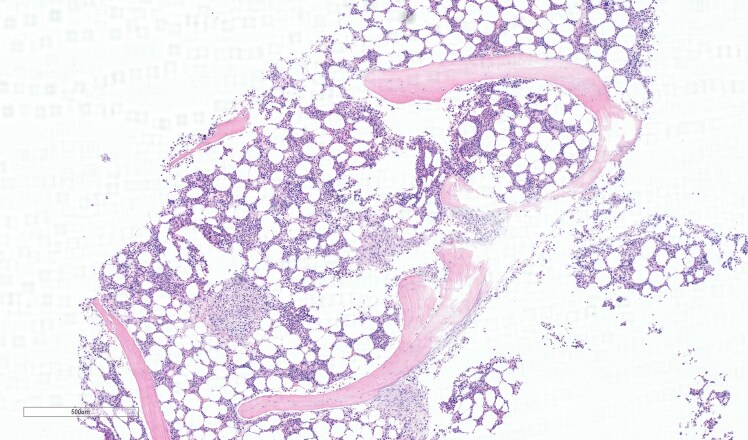
Bone marrow biopsy (haematoxylin and eosin, total magnification ×4; scale bar = 500 µm) staining showing clusters of atypical mast cells.

**Figure 6:**
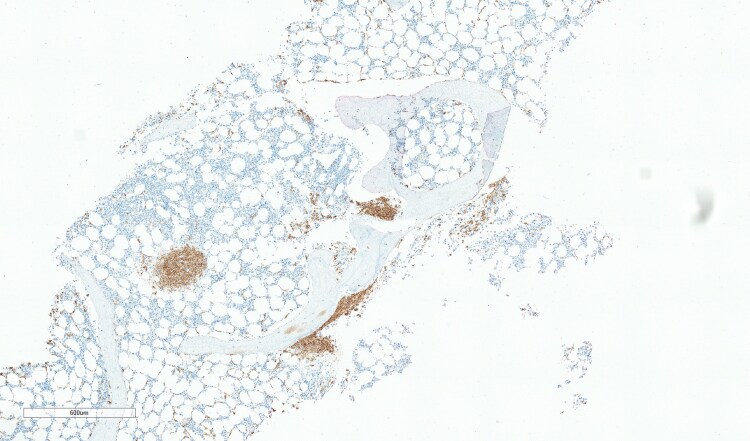
Skin biopsy (CD117 stain, total magnification ×3.8; scale bar = 600 µm) showing CD117 positively stained mast cells (brown stained), a marker of mast cell disease. This confirms systemic mastocytosis rather than cutaneous mastocytosis.

The patient gives an account of his life with systemic mastocytosis (SM). Mastocytosis results from a clonal neoplastic proliferation of mast cells in both cutaneous and extracutaneous organs including bone marrow (Figure 5), spleen and lymph nodes. Adult onset usually occurs between the ages of 20 and 40 years, with a prevalence of 1/10 000.^1^ Cutaneous presentations include maculopapular (urticaria pigmentosa), diffuse cutaneous mastocytosis and mastocytoma of the skin (Figure 6).^2^

SM is an aggressive form of the disorder characterized by the release of vasoactive cell mediators due to excessive activity of mast cells, which can result in a variety of symptoms.^3^ Like many patients, this patient suffers with intractable, unrelenting itch. In previous studies, cutaneous involvement was seen in 79.1% of patients with SM.^3^

As described above, symptoms have a profound impact on patients’ quality of life, mental health and cognitive function. More than half of individuals with mast cell disorders in one study found their illness very stressful, and nearly a third experienced moderate levels of anxiety.^3,4^

Indolent systemic mastocytosis (ISM) is a clonal mast-cell disease driven by the KIT D816V mutation. Early trials have shown that avapritinib, a receptor tyrosine kinase inhibitor, was superior to placebo in reducing uncontrolled symptoms and mast-cell burden in patients with ISM.^5^ It is currently registered by the European Commission for treatment of patients with aggressive systemic mastocytosis, after at least one systemic therapy.

## Data Availability

The data underlying this article are available in the article.
